# The proteome of perilymph in patients with vestibular schwannoma. A possibility to identify biomarkers for tumor associated hearing loss?

**DOI:** 10.1371/journal.pone.0198442

**Published:** 2018-06-01

**Authors:** Jesper Edvardsson Rasmussen, Göran Laurell, Helge Rask-Andersen, Jonas Bergquist, Per Olof Eriksson

**Affiliations:** 1 Department of Surgical Sciences, Otorhinolaryngology and Head and Neck Surgery, Uppsala University, Uppsala, Sweden; 2 Department of Chemistry – BMC, Analytical Chemistry, Uppsala University, Uppsala, Sweden; Pacific Northwest National Laboratory, UNITED STATES

## Abstract

**Background:**

Due to the surrounding bone, the human inner ear is relatively inaccessible and difficult to reach for cellular and molecular analyses. However, these types of investigations are needed to better understand the etiology, pathophysiology and progression of several inner ear disorders. Moreover, the fluid from the inner ear cannot be sampled for micro-chemical analyses from healthy individuals *in vivo*. Therefore, in the present paper, we studied patients with vestibular schwannoma (VS) undergoing trans-labyrinthine surgery (TLS). Our primary aim was to identify perilymph proteins in patients with VS on an individual level. Our second aim was to investigate the proteins identified at a functional level and our final aim was to search for biological markers for tumor-associated hearing loss and tumor diameter.

**Methods and findings:**

Sixteen patients underwent TLS for sporadic VS. Perilymph was aspirated through the round window before opening the labyrinth. One sample was contaminated and excluded resulting in 15 usable samples. Perilymph samples were analyzed with an online tandem LTQ-Orbitrap mass spectrometer. Data were analyzed with MaxQuant software to identify the total number of proteins and to quantify proteins in individual samples. Protein function was analyzed using the PANTHER Overrepresentation tool. Associations between perilymph protein content, clinical parameters, tumor-associated hearing loss and tumor diameter were assessed using Random Forest and Boruta. In total, 314 proteins were identified; 60 in all 15 patients and 130 proteins only once in 15 patients. Ninety-one proteins were detected in at least 12 out of 15 patients. Random Forest followed by Boruta analysis confirmed that alpha-2-HS-glycoprotein (P02765) was an independent variable for tumor-associated hearing loss. In addition, functional analysis showed that numerous processes were significantly increased in the perilymph. The top three enriched biological processes were: 1) secondary metabolic processes; 2) complement activation and 3) cell recognition.

**Conclusions:**

The proteome of perilymph in patients with vestibular schwannoma has an inter-individual stable section. However, even in a cohort with homogenous disease, the variation between individuals represented the majority of the detected proteins. Alpha-2-HS-glycoprotein, P02765, was shown to be an independent variable for tumor-associated hearing loss, a finding that needs to be verified in other studies. In pathway analysis perilymph had highly enriched functions, particularly in terms of increased immune and metabolic processes.

## Introduction

Due to the surrounding bone, the human inner ear is relatively inaccessible and difficult to reach for cellular and molecular analyses. However, such investigations are needed in order to gain a better understanding of the etiology, pathophysiology and progression of several inner ear disorders. The inner ear harbors the membranous system, consisting of the cochlear and semicircular ducts with ampullas, the otolith organs (saccule and utricle) and the endolymphatic duct and sac. The labyrinth membrane separates the two extracellular inner ear fluids, referred to as perilymph and endolymph. Endolymph is rich in potassium, and in the cochlea, is also associated with an electric field potential (the endo-cochlear potential or EP) which is essential for hair cell transduction and hearing [1,2].

For a variety of reasons, samples of inner ear fluid cannot be obtained for micro-chemical analyses from healthy individuals *in vivo*. However, the pioneers of proteomic analysis in perilymph, Arrer et al. [3], analyzed post-mortem perilymph, cerebrospinal fluid (CSF) and serum samples and revealed different patterns of α1-antitrypsin and pre-albumin, thus suggesting that perilymph is produced within the inner ear. Another study examined perilymph from patients with perilymph fistula and found 100 different proteins [4]. Of these, 30 proteins were identified and quantified; these authors used Sodium dodecyl sulfate polyacrylamide gel electrophoresis or two-dimensional polyacrylamide gel electrophoresis to separate proteins, followed by Western blot antibody staining for identification purposes.

Advancements in mass spectrometry (MS) for the identification of proteins have greatly improved the sensitivity, dynamic range and reproducibility of protein identification. MS was first applied to perilymph by Lysaght et al. who identified another 42 proteins in samples from patients undergoing surgery for vestibular schwannoma (VS) or cochlear implantation [5].

At our institution, we use two surgical techniques for removal of skull-base tumors: trans-labyrinthine (TLS) and transcochlear surgery (TCS). During surgery aspiration of perilymph can be performed either through a cochleostomy, or through the round window membrane (RWM). However, a general problem is to acquire samples of perilymph that are free from contamination. Even in experimental models using optimal scientific design, CSF and blood impurity remains a significant technical challenge [3].

In the present study, we studied patients with VS who were undergoing TLS. VS is a benign tumor arising from the VIIIth cranial nerve, with an overall incidence of approximately 1 per 100 000 individuals [6]. The most common presenting symptom of VS is an asymmetric loss in sensorineural hearing [7]. At the time of presentation, the growth and progression of symptoms are often difficult to predict. Depending on tumor diameter, a wait-and-scan principle may be adopted [8]. Generally, treatment options include radiation therapy or surgical removal to decompress the brainstem and cerebellum.

The primary aim of this study was to identify perilymph proteins in patients with VS on an individual level. Other aims were to investigate identified proteins at a functional level and search for biological markers for tumor-associated hearing loss and tumor diameter.

## Patients and methods

### Patients

Sixteen patients undergoing TLS for sporadic VS, with varying degree of sensorineural hearing loss, were included in this study. Surgery was performed at the Department of Neurosurgery, Uppsala University Hospital, Sweden. Post-operatively, all tumors were confirmed by histological analysis as VS. Patients with previous neurosurgery were excluded, as were those with comorbidity.

Two filters were employed to exclude perilymph samples from accidental blood contamination. First, an optical inspection and comparison of color was made prior to mass spectrometry. Secondly, samples presenting with a high albumin signal, combined with a low total number of proteins, were considered to be contaminated with blood [3], and were therefore excluded from further proteomic analysis. In total, 16 patients were included, but the sample from one patient was excluded after MS, resulting in the final study involving 15 patients.

Tumor-associated hearing loss was defined as the difference between the pure tone average (PTA 4), at thresholds of 500, 1000, 2000, 4000 Hz in the affected and unaffected ear. Tumor diameter was measured on the last preoperative magnetic resonance imaging (MRI) and defined as the greatest extra meatal diameter (Table 1).

**Table 1:** Clinical parameters of patients included in the study.

Gender	PTA4 affected side in dBHL	Tumor associated hearing loss in dBHL	Tumor Ø mm
Male	48	45	17
Male	24	17	19
Male	52	47	21
Male	13	8	17
Male	61	28	40
Female	15	10	18
Female	29	10	6
Male	51	20	24
Male	60	57	27
Female	26	8	40
Female	34	34	18
Female	61	53	28
Male	110	100	14
Male	81	73	21
Female	73	39	16

dBHL, decibel hearing level; PTA4, Pure tone average at 500, 100, 2000, 4000 Hz; Tumor associated hearing loss in dBHL was defined as the difference of PTA4 between the affected and unaffected ears; Tumor Ø mm, Tumor diameter defined as the greatest extra meatal diameter on preoperative magnetic resonance imaging.

### Sampling of perilymph

Perilymph was aspirated at TLS through the RWM after removal of the posterior bony ear canal wall. This was combined with removal of the lateral ossicles, ear drum, and ear canal skin, closure of the external auditory canal skin and sealing of the Eustachian tube. This procedure has reduced the risk of postoperative rhino-liquorrhea at our center [9,10]. All patients were given anti-thrombotic dalteparin, subcutaneous injection of 2500 international units, on the morning of the day of surgery, or after the placement of external ventricular drainage when this was indicated. Before sampling, we ensured careful hemostasis and cleaning of the surgical field to reduce blood contamination. One perilymph sample was taken from each patient. The RWM was perforated with a sharp needle and an Eppendorf Research^®^-Plus Variable pipette (5–10 μL, Eppendorf AG, Hamburg, Germany) with a 20 μL tip was used to aspirate a maximum of 10 μL perilymph during a period of approximately 10 seconds. The sample was immediately frozen in liquid nitrogen and later transferred to -80°C. All samples were collected using sterile technique, including sterile vials for the fluid samples.

### Preparation for mass spectrometry

Samples were freeze-dried over night to remove aqueous content and dissolved in Sodium Dodecyl Sulfate lysis buffer (4% SDS in 100 mM Tris/HCl pH 7.6) at 70°C for 5 minutes. Solubilized proteins were separated from sample debris by centrifugation at 16,000 g for 5 minutes, and protein concentration was determined by a detergent compatible protein assay (Bio-Rad, Hercules, USA). Enzymatic fragmentation of proteins was performed by filter-aided sample preparation [11]. In brief, protein disulfide bonds were reduced with 100 mM dithiothreitol at 37°C for 30 minutes prior to sample loading onto a 30 kDa centrifugal filter unit (Millipore, Merck, Germany). Next, samples were washed with urea buffer (8 M urea in 100 mM Tris/HCl pH 8.5) and alkylated with 550 mM iodoacetamide. Protein digestion was performed by Lys-C (protein-to-enzyme ratio 100:1) (Wako Chemicals GmbH, Japan) at 30°C for 2 hours, followed by Trypsin (protein-to-enzyme ratio 100:1) (Promega Corporation, USA) at 37°C overnight. The resulting peptides were eluted with 50 mM tetraethylammonium bromide, acidified, and dried before further processing. For semi-quantitative proteome analysis, a label-free strategy was used.

Reverse-phase liquid chromatography for peptide separation was performed using an Easy Nano Flow System (Thermo Fisher Scientific) coupled to an LTQ-Orbitrap Velos Pro mass spectrometer (Thermo Fisher Scientific). Peptides were separated by a pre-column (100 μm ID, 5 μm C18-beads) and analytical columns (75 μm ID, 3 μm C18-beads) (Thermo Fisher Scientific), using a linear gradient from 4% to 48% acetonitrile with 0.1% formic acid for 221 min at a flow rate of 250 nl/min, followed by 75% acetonitrile for 10 min and 4% acetonitrile for 9 min for re-equilibration. After separation, peptides were ionized using a nano-electrospray ionization source and transferred into the mass spectrometer. Full survey scan spectra (m/z = 400–1750) were acquired in the Orbitrap with a resolution of 60,000 after accumulation of 1,000,000 ions. The 10 most intense peaks were isolated and fragmented in the linear ion trap using collision-induced dissociation (35% normalized collision energy). The mass spectrometer was used in a data-dependent mode.

### Proteomic analysis

MS data were analyzed using Proteome Discovery 2.1.0.81, supplied by Thermo Scientific, to identify the total number of proteins. MaxQuant 1.5.6.5, supplied by Max Plank institute, was used to quantify the proteins in individual samples using the Andromeda search engine to correlate MS/MS spectra to the Uniprot human database [12]. The following parameters were used for data processing: maximum of two miss cleavages, mass tolerance of 4.5 ppm for main search, trypsin as digesting enzyme, carbamidomethylation of cysteins as fixed modification, and the oxidation of methionine and acetylation of the protein N-terminus as variable modifications. Only peptides with a minimum of 7 amino acids, as well as at least one unique peptide, were required for protein identification. Only proteins with at least two peptides, and at least one unique peptide, were considered as being identified and used for further data analysis.

### Pathway analysis

The PANTHER Overrepresentation Test, released 2016-07-15, with annotation version 11.1, released 2016-10-24, was used to identify all up- or down-regulated pathways [13]. This method compares proteins within a sample with a reference of choice, for example, the human genome. The reference used was the whole *Homo sapiens* genome (reference list 20972). This was followed by an over-representation test that presents up- or down-regulations of proteins in a selected sample. We choose to present only significant (p<0.05) up- or down-regulated pathways in our results.

### Statistical methods

Analysis was designed to assess the associations between perilymph protein content and clinical parameters, tumor-associated hearing loss and tumor diameter. Only proteins detected in at least 12 out of the 15 samples were included in the final analysis. This cut off was decided upon after analyzing the detection frequency for each protein in the samples, to ensure that there was consistency in terms of missing values. First, we applied univariate linear regression with hearing loss or tumor diameter as dependent variables, and protein levels as explanatory variables. This allowed us to create an overview of the data. Random Forest was used to determine the variable importance of all variables in the data [14] followed by the Boruta method, which is based on repeated Random Forest analyses, to evaluate if the result was independent from random variations [15].

### Ethic statements

This study was approved by the regional ethics committee in Uppsala (ref 2013/255). Oral and written consent was obtained from all patients prior to surgery and the study complied with the rules of the Declaration of Helsinki.

## Results

### Quantification of perilymph proteins

**Fig 1:**
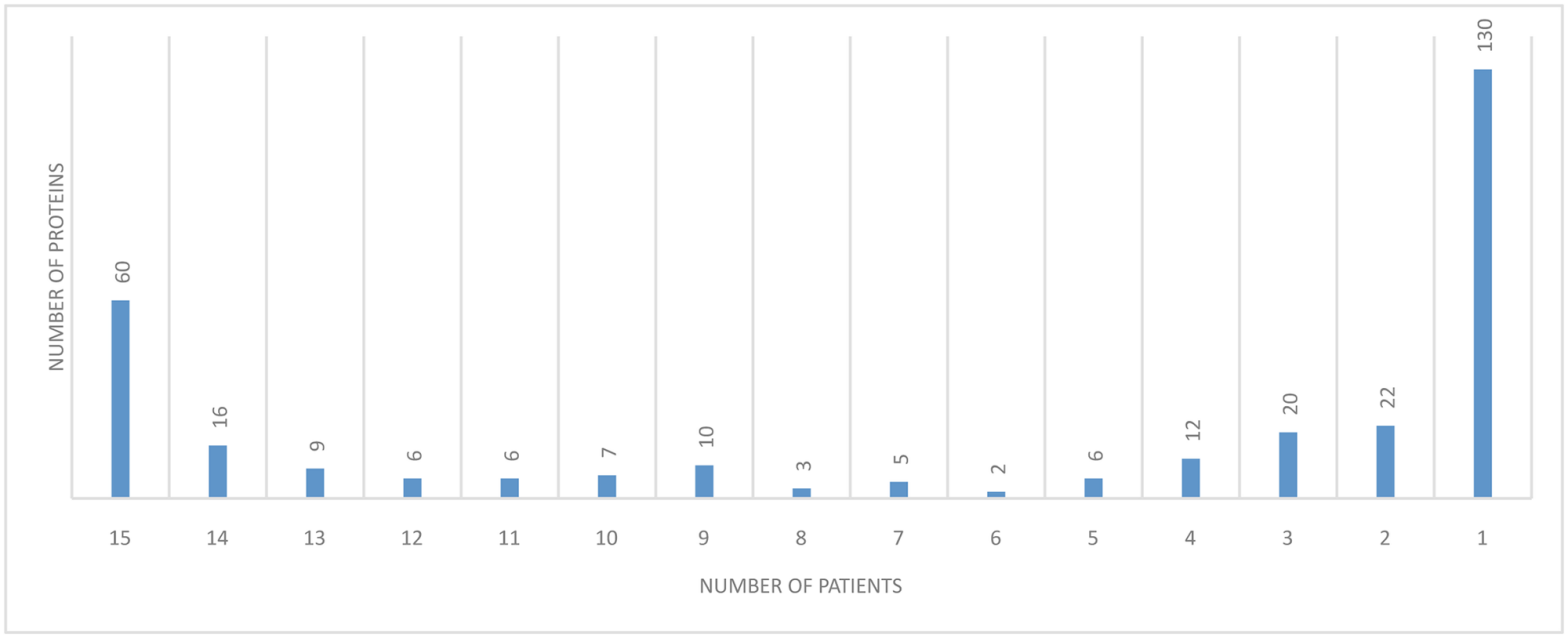
The number of proteins identified in declining order of patients. This figure shows the distribution of 314 proteins identified in perilymph samples from fifteen patients. Sixty proteins were identified in all samples, and 91 in 12 or more samples. Note that 130 proteins were only identified once in 15 patients. Mass spectrometer data was analyzed using MaxQuant software.

In total, MaxQuant identified 314 different proteins; 60 of these proteins were identified in all 15 patients and 130 proteins were only identified once in 15 patients. Ninety-one proteins were detected with a cut-off set to 12 out of 15 patients, and hereafter referred to as ‘frequently identified proteins’. In total, 184 proteins were found in four perilymph samples or less (Fig 1). The full list of proteins identified by MaxQuant is provided in supporting information S1 Table.

### Perilymph proteins and tumor-associated hearing loss

Of the 91 frequently-identified proteins in the perilymph, four proteins were shown by univariate linear regression to be significantly correlated to tumor-associated hearing loss: Ig gamma-4 chain C region (P01857; p = 0.005); Ig kappa chain C region (P01834; p = 0.015); Complement C3 (P01024; p = 0.016) and immunoglobulin heavy constant gamma 3 (P01860; p = 0.023) (Table 2).

**Table 2:** Univariate linear regression results for proteins vs. tumor-associated hearing loss.

Protein Ids	Estimate	95% CI	*p* value	Number of samples in which the protein was identified
P01857	18.16	(7.5, 28.83)	0.0053	15
P01834	16.43	(4.97, 27.9)	0.0148	15
P01024	16.3	(4.78, 27.82)	0.0158	15
P01860	15.51	(3.68, 27.35)	0.0234	15

P01857, Ig gamma-4 chain C region; P01834, Ig kappa chain C region; P01024, Complement C3; P01860, immunoglobulin heavy constant gamma 3; 95% CI, 95% confidence intervals.

**Fig 2:**
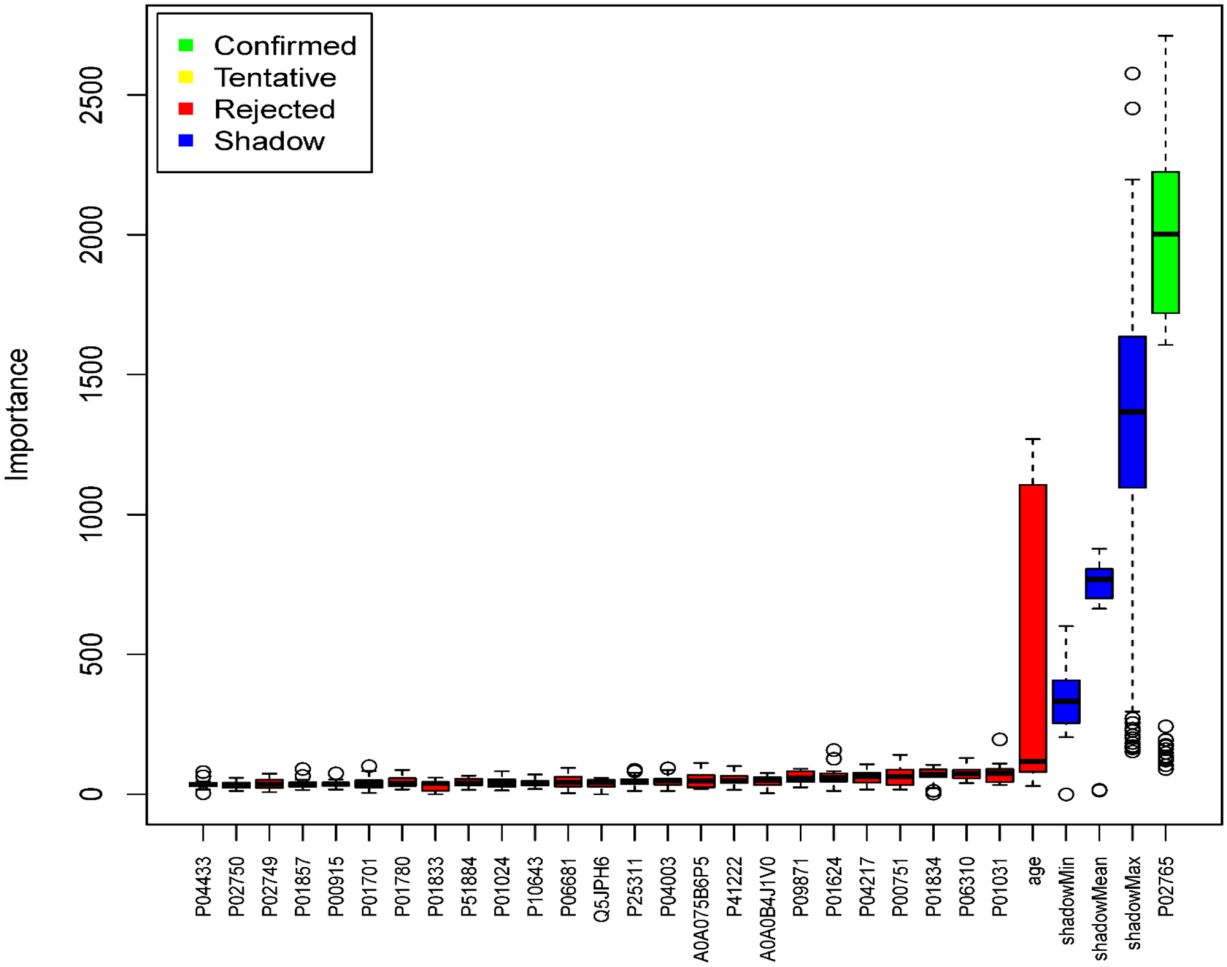
Boruta analysis for the outcome tumor-associated hearing loss. In brief Boruta analysis identifies variables performing better than random variables based on repeated Random Forest analysis. The shadow value represents random data. Boruta analysis showed that Alpha-2-HS-glycoprotein (P02765) was stronger than random data, while the four proteins identified as significant by univariate linear regression (P01857, P01834, P01024 and P01860) were rejected.

Random Forest and Boruta methodology, confirmed that Alpha-2-HS-glycoprotein (P02765) was an independent variable for tumor-associated hearing loss. Alpha-2-HS-glycoprotein was identified in all patients. However, the four proteins shown to be significant by univariate linear regression (P01857, P01834, P01024 and P01860) were rejected by Boruta analysis, showing that these could represent false positive results (Fig 2).

### Perilymph proteins and tumor diameter

Univariate linear regression showed that tumor diameter was correlated with the expression of N-acetylmuramoyl-L-alanine amidase (Q96PD5; 95%CI: 0, 46–8, 94; p = 0.049). However, Random Forest followed by Boruta graded complement component C6 (P13671) as "tentative" independent variable for tumor diameter, and rejection of Q96PD5.

### Pathway analysis of perilymph

To better understand the functional roles of perilymph proteins, we applied the PANTHER overrepresentation test. This test used the protein list from Proteome Discovery, which identified 312 different proteins in the 15 samples of perilymph. Thirty-three proteins were variations of highly variable immunoglobulin sites, and from a pathway perspective, these were considered as variations of only one protein and were therefore excluded in further analysis. In total, 279 unique proteins were included in the PANTHER overrepresentation test [13]. The functions of the 279 perilymph proteins were classified into five main categories according to Gene Ontology (GO) [16] combined with two PANTHER subsets of ontologies: 1) biological process; 2) molecular function and 3) cellular component, 4) PANTHER Protein Class and 5) PANTHER Pathways. These five classes are referred to as ‘PANTHER-GO-slim’ ontologies [17]. Results are shown in logarithmic graphs to provide a comprehensive overview of the significant up- and down-regulated processes within the perilymph.

### Biological process

**Fig 3:**
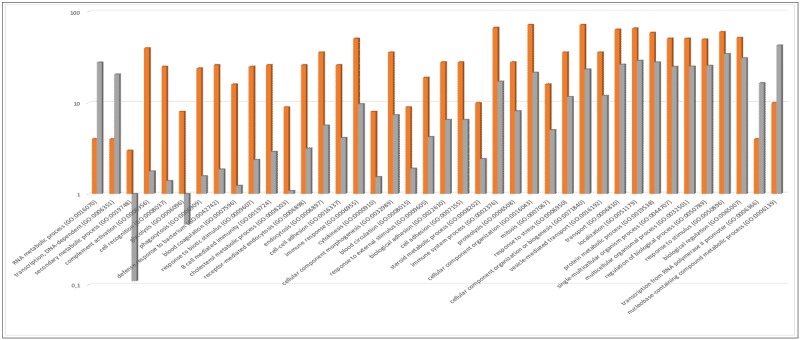
PANTHER overrepresentation test of biological processes. Orange bars represent the proteins detected in the perilymph while gray bars represent the expected number of proteins in each biological process group. The scale is logarithmic. In total, perilymph proteins were involved in 38 up-regulated biological processes while involved in two down-regulated biological processes.

The biological process classification describes the biological system that a protein contributes to within the cell or organism. For example, the insulin receptor contributes to regulate the processes involved in carbohydrate metabolism [16]. The identified perilymph proteins were involved in 40 significantly up- or down-regulated biological processes. Of these, 38 were up-regulated and two were down-regulated. The three most up-regulated processes were secondary metabolic processes, complement activation and cell recognition. RNA metabolic processes and transcription DNA-dependent processes were the only process which was down regulated (Fig 3). Full data are given in supporting information S2 Table.

Alpha-2-HS-glycoprotein (P02765) is part of the acute-phase response (GO:0006953), neutrophil degranulation (GO:0043312) and pinocytosis (GO:0006907), which all have ancestry links to highly up-regulated biological processes [18].

Acute-phase response (GO:0006953) is a response to stress (GO:0006950) (18) and was enriched by 3.09-fold (p = 6.64 x 10^−7^) in perilymph compared to the expected level. This process is also related to complement activation (GO:0006956) which was enriched by 22.55-fold (p = 3.38 x 10^−38^), and immune response (GO:0006955), which was enriched by 5.25-fold (p = 1.04 x 10^−19^)[18].

Neutrophil degranulation (GO:0043312) is part of immune response (GO:0006955)[18] which was enriched by 5.25-fold (p = 1.04 x 10^−19^) while pinocytosis (GO:0006907) is a form of endocytosis (GO:0006897) and was enriched by 6.36-fold (p = 6.35 x 10^−16^)[18].

### Molecular function

**Fig 4:**
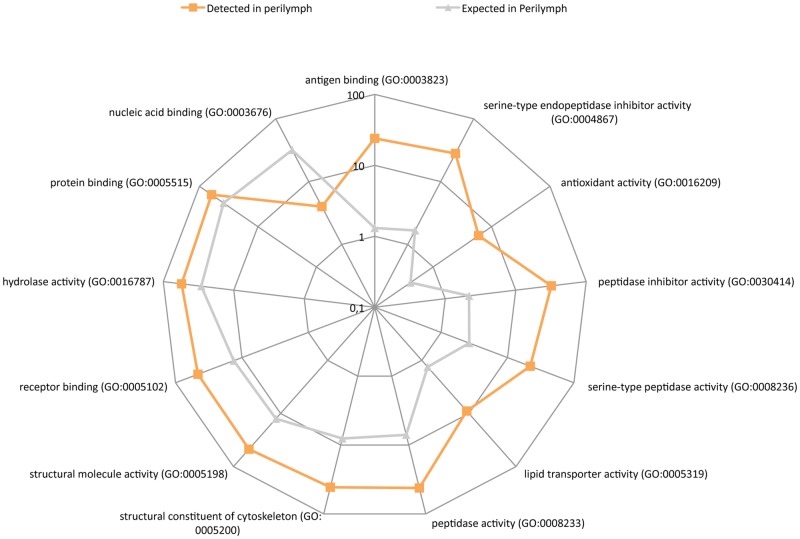
PANTHER overrepresentation test of molecular function. Radar diagram, with a logarithmic scale, showing a comparison between the numbers of expected and detected proteins in each molecular function group. Orange boxes represent the detected number while gray triangles show the expected number. In total, 12 enriched molecular functions and one down-regulated.

Molecular function refers to the functional interaction a protein carries out with its molecular targets. For example, the insulin receptor exhibits transmembrane tyrosine kinase activity, and can add a phosphate group to a tyrosine in another protein [16]. Molecular function analysis revealed 13 enriched functions. The most highly up-regulated molecular functions were antigen binding, serine-type endopeptidase inhibitor activity, antioxidant activity and peptidase inhibitor activity (Fig 4). Please refer to supporting information S3 Table for the full dataset.

Alpha-2-HS-glycoprotein (P02765) is a direct part of endopeptidase inhibitor activity (GO:0004866) and cysteine-type endopeptidase inhibitor activity (GO:0004869). These groups prevent or reduce the activity of an endopeptidase or cysteine-type endopeptidase, a form of enzyme that hydrolyzes non-terminal peptide bonds. The closely related molecular function serine-type endopeptidase inhibitor activity (GO:0004867) was enriched by 16.8-fold (p = 5.43 x 10^−23^) [18].

### Cellular component

**Fig 5:**
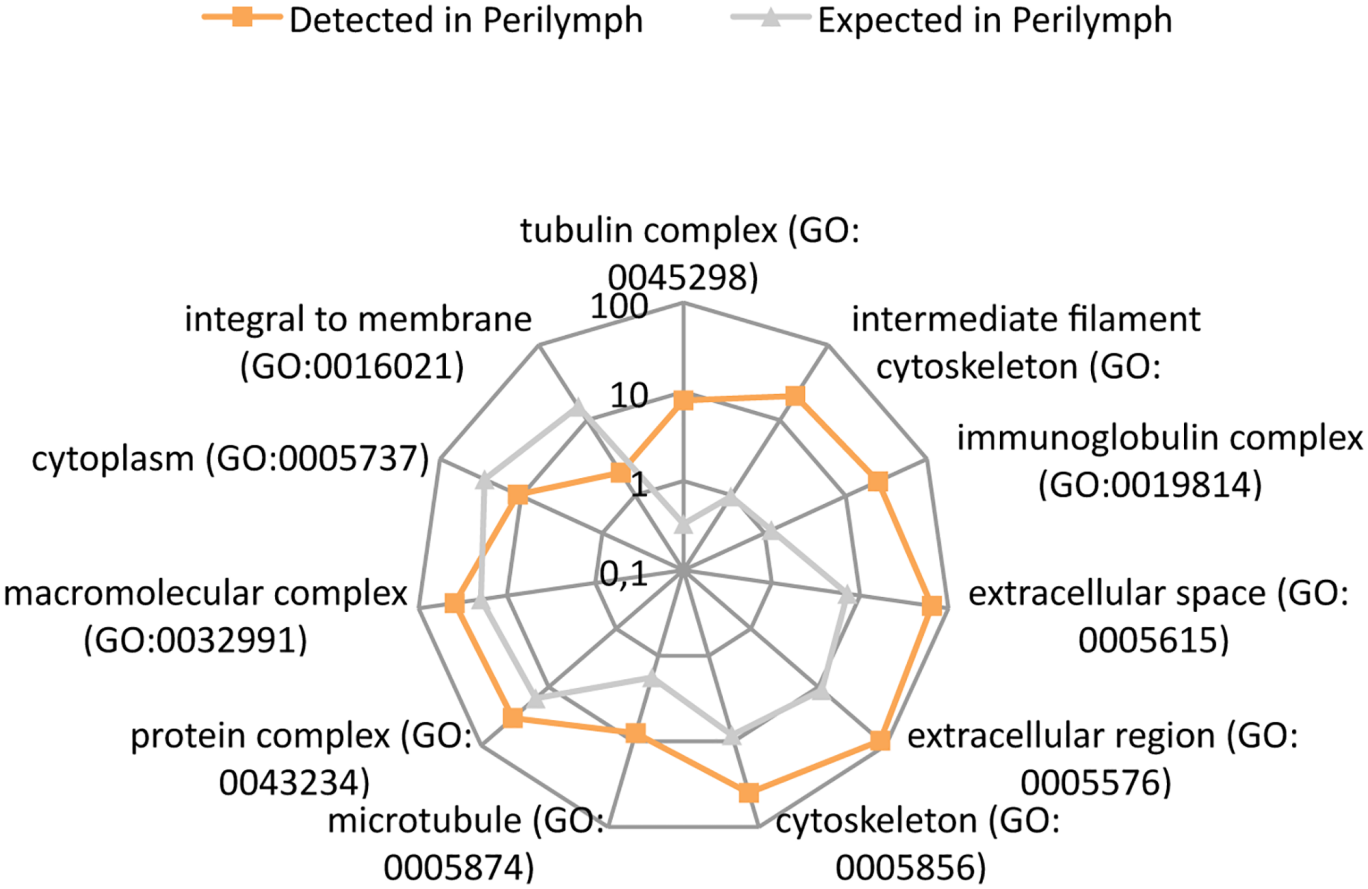
PANTHER overrepresentation test of cellular component. Radar graph with a logarithmic scale showing a comparison between the numbers of expected and detected proteins in each cellular component group. Within the perilymph (orange boxes), nine functions were up-regulated compared to expected levels (gray triangles) while two were down-regulated.

Cellular component ontology includes subcellular structures, such as the nuclear membrane, and refers to the location of a protein, and where it performs its functional activity. For example, the insulin receptor is located in the plasma membrane [16]. The most highly enriched cellular components found in perilymph were tubulin complex, intermediate filament cytoskeleton and immunoglobulin complex. Only the ‘integral to membrane’ group was down-regulated (Fig 5). Please refer to supporting information S4 Table for a complete dataset.

In the cellular component ontology, alpha-2-HS-glycoprotein (P02765) was related to the extracellular region (GO:0005576) and was enriched by 7.37-fold (p = 1.57 x 10^−45^). These data implies that this protein is found outside of the plasma membrane and is active outside of the cell [18].

### PANTHER pathways

**Fig 6:**
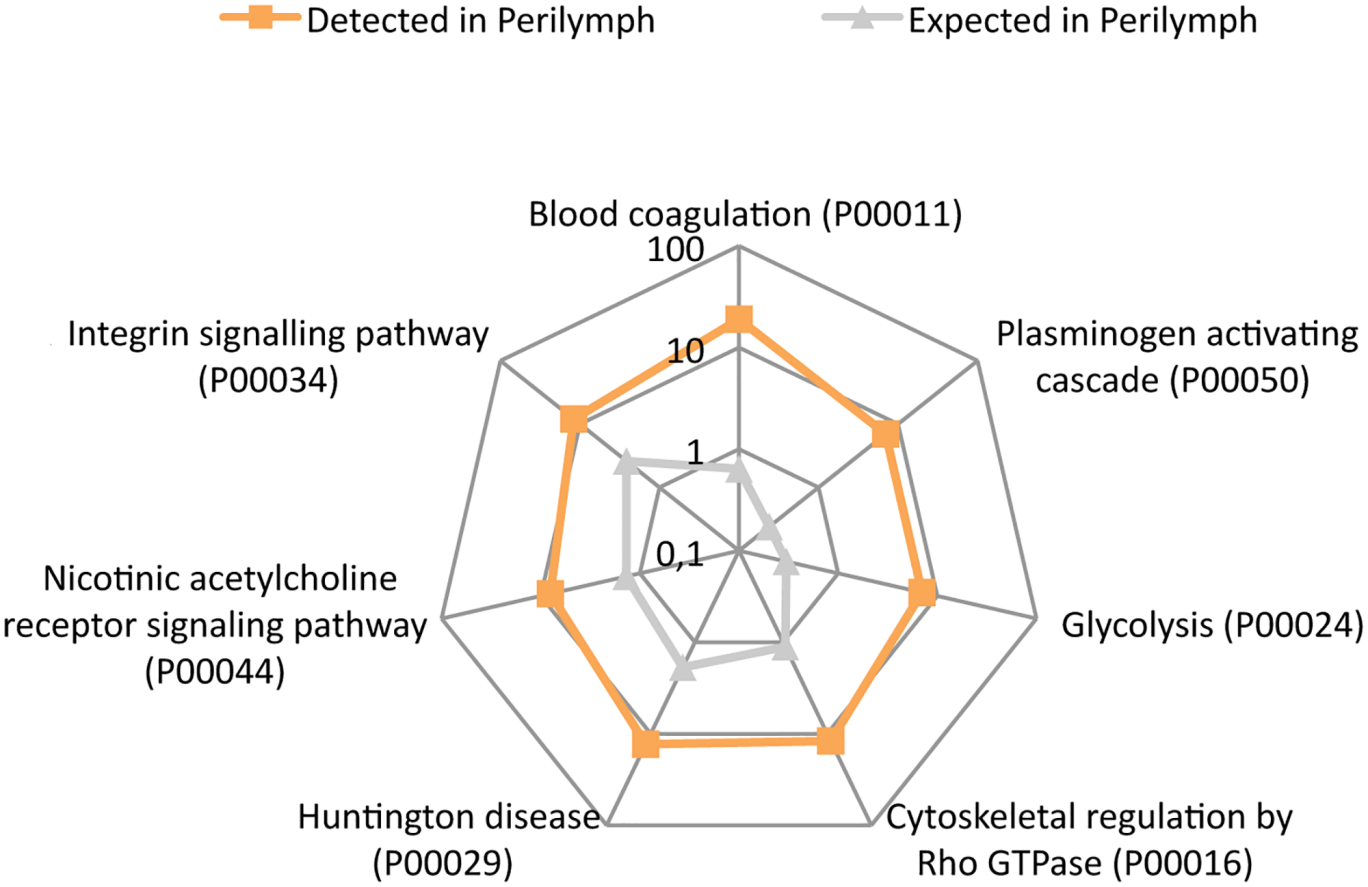
PANTHER overrepresentation test of PANTHER pathways. Image shows a logarithmic radar graph comparison between the detected (orange boxes) and expected (gray triangles) numbers of proteins in each group. Seven significantly up-regulated PANTHER pathways were found.

PANTHER pathways analysis is similar to biological process analysis, but also specifies the relationship between interacting molecules; for example, well known models for a process in a cell or tissue which includes some functions not covered by GO biological process [17]. PANTHER pathway analysis revealed 7 significantly up- or down-regulated pathways. The most enriched pathways were blood coagulation, the plasminogen activity cascade, and glycolysis (Fig 6). Please refer to supporting information S5 Table for a complete dataset.

### PANTHER protein class

**Fig 7:**
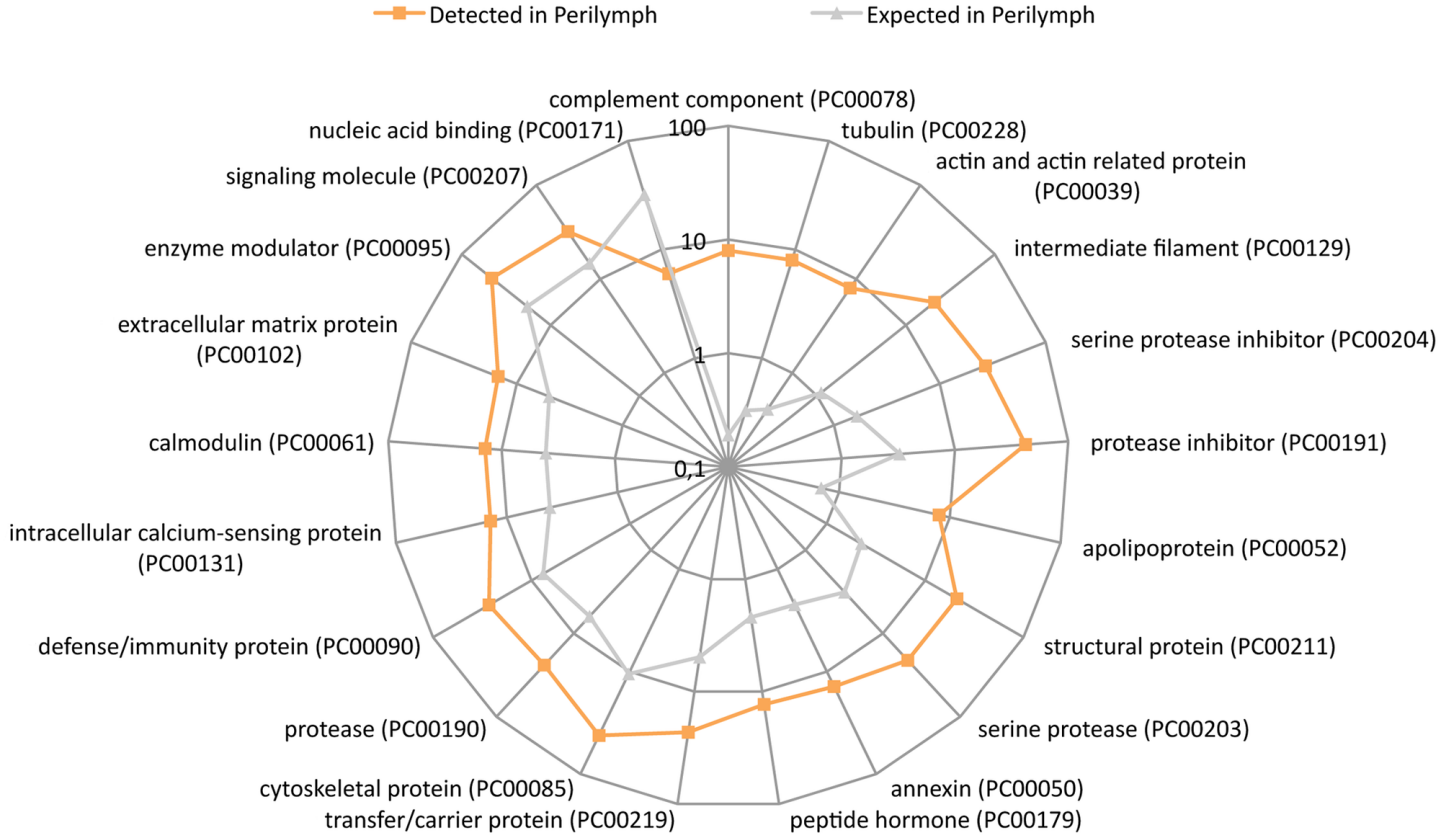
PANTHER overrepresentation test of PANTHER protein class. Image shows a logarithmic radar graph of the comparison between the number of detected (orange boxes) and expected (gray triangles) proteins in each group. Twenty up-regulated protein classes and one down-regulated were identified.

PANTHER protein class analysis is similar to GO molecular function analysis but is an adaptation of earlier PANTHER ontology. This includes several protein classes that are not included in the GO molecular function ontology [17]. Twenty protein classes were up-regulated and only one was down-regulated. The most highly enriched PANTHER protein classes were complement component, tubulin, actin and actin-related proteins. Only nucleic acid binding was down-regulated (Fig 7). Please refer to supporting information S6 Table for a complete dataset.

## Discussion

Modern surgical approaches, combined with advances in analytical techniques, have provided us with new opportunities with which to explore perilymph in the inner ears of humans affected by different pathological conditions. This study was performed exclusively on patients with VS to characterize the perilymph in these patients at an individual level. We did this by applying MS-technology for proteomic analysis. The individual approach provided new insight of the large variations in the protein content of perilymph in VS disease. With a uniform diagnosis, it was also possible to identify inter-individual differences related to clinical outcome. No sampling technique is currently available that allows sampling of perilymph *in vivo* from individuals with normal inner ear function. Therefore, studies of the normal perilymph proteome are not yet possible.

Sampling of perilymph is technically difficult, and several previous studies have reported problems with blood contamination. In the present study, careful micropipette aspiration through the RWM, before opening the labyrinth, resulted in a low incidence of contaminated samples. The same surgical approach, but with a different sampling material and technique, was also reported in another study [19]. Our outcome was very favorable, with only 1 out of 16 samples contaminated, compared to 16 out of 24 samples in a previous report [3].

The total number of identified proteins in the present study (314) is consistent with earlier reports using non-selective approaches to analyze the proteome of perilymph from patients with differential inner ear diagnosis at a group level [4][5][19]. Furthermore, 91 proteins were identified in 12 to 15 samples, and this was validated by comparison with data arising from previous MS studies on perilymph [5,19]. Eighty-nine of these frequently identified proteins were also found in samples from VS patients in two earlier studies and can be presumed to represent a stable part of the perilymph proteome in patients with VS. Immunoglobulin heavy variable 3–15 and Ig kappa chain V-II region FR, variations on immunoglobulins, are the two proteins that were not previously identified. The list of stipulated normal proteome in perilymph [5] were compared to our results, and 51 out of 71 could be found among the frequently detected proteins in perilymph from VS patients. This discrepancy could be explained by the large inter individual variation demonstrated here with 184 out of 314 proteins detected in 4 patients or less, even with a homogenous diagnosis group. This variation is important to take into account for in future inner ear studies.

To date, there have been no methods developed to predict hearing loss or tumor growth in patients with VS. This would be of significant value in the planning of clinical management. The present work used two estimates to study these clinically important variables. The difference in PTA between the affected and unaffected ear was used as an estimate of tumor-associated hearing loss and tumor diameter was considered as an estimate of tumor growth.

The progression of hearing loss may depend on several factors such as the origin of the tumor along the vestibular nerve, the site of auditory nerve compression, the tumors tendency to infiltrate nerve axons and excretions from the tumor. The mechanisms are mostly unknown, therefore characterization of the perilymph proteome in VS patients may provide a valuable addition to understand the mechanisms of tumor-associated hearing loss. Recently studies of the proteome were applied to investigate various heat shock proteins, and they might be of importance for preservation of residual hearing after cochlear implantation [20]. However, we could not replicate this in our study since we only detected three variations of Heat shock 70KDa proteins in only a single patient.

In contrast to earlier mass spectrometry studies on perilymph [5,19], the present study was based on a homogenous cohort featuring individual analysis of each sample. This provided the possibility of hypothesis-generated statistical methods and allowed us to explore the possible association between protein expression and tumor-associated hearing loss. We hypothesized that the difference in pure tone average thresholds between the affected and unaffected ear could represent hearing loss associated with the VS. Alpha-2-HS-glycoprotein (P02765) was confirmed by nonlinear multivariate methods Random forest and Boruta as an independent variable for tumor-associated hearing loss. The interpretation of the presences of alpha-2-HS-glycoprotein is eluding since it was present in all samples, and the concentration is not directly related to hearing threshold. But the fact it was also found in the samples from VS patients in an study from 2017 [19] encourage to further explore if there is a causative relation.

The lack of samples of perilymph from healthy individuals is an obstacle in mapping the pathological processes found in perilymph of VS patients, therefore an animal model is necessary. Compared with perilymph from patients having cochlear implants in a previous study shows the presence of alpha-2-HS-glycoprotein in many samples[19]. The majority of these patients have unknown cause of sensorineural hearing loss [19], and it cannot be excluded that the alpha-2-HS-glycoprotein could be involved in sensorineural hearing loss even in the absence of a VS.

To understand the functions of the detected proteins, the use of pathway analysis, such as the PANTHER overrepresentation test, provides us with an informative presentation of protein-related activities in the inner ear. In the present study, we presented functional groups, including information relating to up- and down-regulation to present a clearer picture of their functions in perilymph. This method relies on the reported functions of the identified proteins in other tissues, and therefore the presented list of functional groups should be considered as an indicator of their possible roles in the inner ear. The procedure/data-base for this type of analysis is available at http://pantherdb.org/.

Investigating the biological process ontology in perilymph was probably the best method with which to understand the general function. In the top 10 enriched biological processes, three immune responses were present: complement activation, defense response to bacteria and B-cell–mediated immunity. Furthermore, three metabolic processes were identified: secondary metabolic processes, glycolysis and cholesterol metabolic processes. The other four processes identified were cell recognition, phagocytosis, blood coagulation and response to biotic stimulus (Fig 3). This indicated that perilymph is much more active from an immunological and metabolic aspect than previously expected.

From the view of biological processes, alpha-2-HS-glycoprotein is part of the acute-phase response, an ancestor to response to stress and also part of neutrophil degranulation an ancestor to immune response [18], which were both significant up-regulated (Fig 3). This is based on data from other tissues and indicates that alpha-2-HS-glycoprotein is a potential inflammatory and immunological mediator in perilymph. The known molecular functions of alpha-2-HS-glycoprotein are as an endopeptidase inhibitor, cysteine-type endopeptidase inhibitor and kinase inhibitor. These groups of inhibitors prevent or reduce the activity of enzymes that hydrolyze non-terminal peptide bonds or catalyze the transfer of a phosphate group to a substrate molecule [18]. As described by cellular component ontology, Alpha-2-HS-glycoprotein is found in the extracellular region [18].

Hence, our hypothesis is that alpha-2-HS-glycoprotein may be excreted from the VS into the extracellular fluid perilymph where it exerts pro inflammatory activity that contributes to sensorineural hearing loss. An alternative hypothesis is that factors derived from the VS influence the inner ear and induce the up-regulation of alpha-2-HS-glycoprotein in perilymph.

The feasibility of using perilymph proteome as an approach for identifying biomarkers of VS is attractive. Our present study identified alpha-2-HS-glycoprotein as an independent factor for hearing loss and since it was part of the stable section of the proteome an interesting candidate as possible biomarker. However, further research is now needed to evaluate in an experimental model if alpha-2-HS-glycoprotein has a pathophysiological role in the hearing loss, and if it can be used as a biomarker to facilitate the decision-making process when choosing between “wait and scan” strategies or surgery.

## Conclusion

Using MS to analyze perilymph from 15 individual patients with VS, we found a stable section of the proteome consisting of 91 proteins which were detected in the majority of patients. The stable section was validated by comparison to earlier proteomic studies on perilymph and 89 proteins were confirmed. There was also a highly individual section of the proteome consisting of 184 proteins only detected in a minority of patients. This showed an important variation in the proteome between individuals, even in a cohort with a homogenous diagnosis.

Furthermore, Random Forest and Boruta analysis showed that alpha-2-HS-glycoprotein, P02765, was an independent variable for tumor-associated hearing loss. This needs to be confirmed by future studies.

Pathway analysis, using the PANTHER overrepresentation test, showed that perilymph exhibited numerous functions which were highly enriched compared to the level expected. Particularly noteworthy was the highly significant increase in the activity of immune and metabolic processes in the perilymph from patients with vestibular schwannoma.

## Supporting information

S1 Table MaxQuant protein identification and quantification. (XLSX)

S2 Table PANTHER overrepresentation test of biological processes. (XLSX)

S3 Table PANTHER overrepresentation test of molecular function. (XLSX)

S4 Table PANTHER overrepresentation test of cellular component. (XLSX)

S5 Table PANTHER overrepresentation test of PANTHER pathways. (XLSX)

S6 Table PANTHER overrepresentation test of PANTHER protein class. (XLSX)

S7 Table Comparison with earlier mass spectrometry studies of the perilymph proteome. (XLSX)

## Abbreviations

CSF: Cerebrospinal fluid
GO: Gene Ontology
MS: Mass spectrometry
PTA4: Pure tone average at 500, 100, 2000, 4000 Hz
RWM: Round window membrane
TCS: Transcochlear surgery
TLS: Translabyrinthine surgery
VS: Vestibular schwannoma

## References

[1] Békésy Gv. DC Resting Potentials Inside the Cochlear Partition. J Acoust Soc Am. 1952;24: 72–76. doi: 10.1121/1.1906851

[2] Tasaki I, Spyropoulos CS. Stria Vascularis as Source of Endocochlear Potential. J Neurophysiol. 1959;22: 149–155. doi: 10.1152/jn.1959.22.2.149 13642091 10.1152/jn.1959.22.2.149

[3] Arrer E, Oberascher G, Gibitz H-J. Protein distribution in the human perilymph: A Comparative Study between Perilymph (Post Mortem), CSF and Blood Serum. Acta Otolaryngol (Stockh). 1988;106: 117–123. doi: 10.3109/00016488809107378 3421092 10.3109/00016488809107378

[4] Thalmann I, Kohut RI, Ryu J, Comegys TH, Senarita M, Thalmann R. Protein profile of human perilymph: in search of markers for the diagnosis of perilymph fistula and other inner ear disease. Otolaryngol—Head Neck Surg Off J Am Acad Otolaryngol-Head Neck Surg. 1994;111: 273–280. 10.1177/01945998941113P117 8084635

[5] Lysaght AC, Kao S-Y, Paulo JA, Merchant SN, Steen H, Stankovic KM. Proteome of Human Perilymph. J Proteome Res. 2011;10: 3845–3851. doi: 10.1021/pr200346q 21740021 10.1021/pr200346q PMC3179892

[6] Kshettry VR, Hsieh JK, Ostrom QT, Kruchko C, Barnholtz-Sloan JS. Incidence of vestibular schwannomas in the United States. J Neurooncol. 2015;124: 223–228. doi: 10.1007/s11060-015-1827-9 26024654 10.1007/s11060-015-1827-9

[7] Kirchmann M, Karnov K, Hansen S, Dethloff T, Stangerup S-E, Caye-Thomasen P. Ten-Year Follow-up on Tumor Growth and Hearing in Patients Observed With an Intracanalicular Vestibular Schwannoma: Neurosurgery. 2016; 1 doi: 10.1227/NEU.0000000000001414 27571523 10.1227/NEU.0000000000001414

[8] Rangel-Castilla L, Russin JJ, Spetzler RF. Surgical management of skull base tumors. Rep Pract Oncol Radiother. 2016;21: 325–335. doi: 10.1016/j.rpor.2014.09.002 27330418 10.1016/j.rpor.2014.09.002 PMC4899518

[9] Ekvall L, Bynke O. Prevention of cerebrospinal fluid rhinorrhea in translabyrinthine surgery. Acta Oto-Laryngol Suppl. 1988;449: 15–16. 10.3109/00016488809106356 3264443

[10] Rask-Andersen H, Kinnefors A, Grängsjö G, Ekvall L. [Translabyrinthine surgery in tumors of the cerebellopontine angle. A suitable technique in large tumors]. Lakartidningen. 1994;91: 2780–2783. 8057733

[11] Wiśniewski JR, Zougman A, Nagaraj N, Mann M. Universal sample preparation method for proteome analysis. Nat Methods. 2009;6: 359 doi: 10.1038/nmeth.1322 19377485 10.1038/nmeth.1322

[12] Cox J, Matic I, Hilger M, Nagaraj N, Selbach M, Olsen JV, et al A practical guide to the MaxQuant computational platform for SILAC-based quantitative proteomics. Nat Protoc. 2009;4: 698 doi: 10.1038/nprot.2009.36 19373234 10.1038/nprot.2009.36

[13] Mi H, Poudel S, Muruganujan A, Casagrande JT, Thomas PD. PANTHER version 10: expanded protein families and functions, and analysis tools. Nucleic Acids Res. 2016;44: D336–D342. doi: 10.1093/nar/gkv1194 26578592 10.1093/nar/gkv1194 PMC4702852

[14] Breiman L. Random Forests. Mach Learn. 2001;45: 5–32. doi: 10.1023/A:1010933404324

[15] Feature Selection with the Boruta Package | Kursa | Journal of Statistical Software. doi: 10.18637/jss.v036.i11

[16] Ashburner M, Ball CA, Blake JA, Botstein D, Butler H, Cherry JM, et al Gene Ontology: tool for the unification of biology. Nat Genet. 2000;25: 25–29. doi: 10.1038/75556 10802651 10.1038/75556 PMC3037419

[17] Mi H, Muruganujan A, Casagrande JT, Thomas PD. Large-scale gene function analysis with the PANTHER classification system. Nat Protoc. 2013;8: 1551–1566. doi: 10.1038/nprot.2013.092 23868073 10.1038/nprot.2013.092 PMC6519453

[18] UniProt: the universal protein knowledgebase. Nucleic Acids Res. 2017;45: D158–D169. doi: 10.1093/nar/gkw1099 27899622 10.1093/nar/gkw1099 PMC5210571

[19] Schmitt HA, Pich A, Schröder A, Scheper V, Lilli G, Reuter G, et al Proteome Analysis of Human Perilymph Using an Intraoperative Sampling Method. J Proteome Res. 2017;16: 1911–1923. doi: 10.1021/acs.jproteome.6b00986 28282143 10.1021/acs.jproteome.6b00986

[20] Schmitt H, Roemer A, Zeilinger C, Salcher R, Durisin M, Staecker H, et al Heat Shock Proteins in Human Perilymph: Implications for Cochlear Implantation. Otol Neurotol. 2018;39: 37–44. doi: 10.1097/MAO.0000000000001625 29227447 10.1097/MAO.0000000000001625

